# Implications of metabolic health status and obesity on the risk of kidney cancer: A nationwide population-based cohort study

**DOI:** 10.3389/fendo.2022.976056

**Published:** 2022-10-20

**Authors:** Yun Kyung Cho, Hwi Seung Kim, Joong-Yeol Park, Woo Je Lee, Ye-Jee Kim, Chang Hee Jung

**Affiliations:** ^1^ Department of Internal Medicine, Hallym University Sacred Heart Hospital, Hallym University College of Medicine, Anyang, South Korea; ^2^ Department of Internal Medicine, Asan Medical Center, University of Ulsan College of Medicine, Seoul, South Korea; ^3^ Asan Diabetes Center, Asan Medical Center, Seoul, South Korea; ^4^ Department of Clinical Epidemiology and Biostatistics, Asan Medical Center, University of Ulsan College of Medicine, Seoul, South Korea

**Keywords:** kidney cancer, metabolic syndrome, obesity, population-based cohort study, phenotypic change

## Abstract

**Purpose:**

This study evaluated the association between metabolic health status and incident kidney cancer among obese participants.

**Materials and methods:**

A total of 514,866 individuals were included from the Korean National Health Insurance Service-National Health Screening Cohort. Changes in metabolic health status and obesity from the baseline examination in 2009–2010 to the next biannual examination in 2011–2012 were determined. Based on the status change, obese participants were divided into four groups: stable metabolically healthy obesity, metabolically healthy obesity to metabolically unhealthy obesity, metabolically unhealthy obesity to metabolically healthy obesity, and stable metabolically unhealthy obesity.

**Results:**

The stable metabolically healthy obesity phenotype did not confer an increased risk of incident kidney cancer, compared to the stable metabolically healthy non-obese group. In contrast, the metabolically healthy obesity to metabolically unhealthy obesity group had a significantly higher risk of incident kidney cancer than the stable metabolically healthy non-obese group. Among patients with metabolically unhealthy obesity at baseline, those who transitioned to the metabolically healthy obese group had no increased risk of kidney cancer, whereas those who remained in metabolically unhealthy obesity status had a higher risk of incident kidney cancer than the stable metabolically healthy non-obese group. The transition or maintenance of metabolic health was a decisive factor for kidney cancer in obese patients.

**Conclusions:**

Maintaining or restoring metabolic health should be stressed upon in obese patients to reduce the risk of kidney cancer.

## Introduction

Obesity leads to several complications and has become a worldwide epidemic in recent decades (1). Comorbidities include the development of cardiometabolic illnesses, such as atherosclerotic cardiovascular diseases, insulin resistance, dyslipidemia, and diabetes, which account for the vast majority of worldwide health issues. More recently, it has been discovered that obesity, particularly severe obesity, is a strong and independent predictor of severe coronavirus disease 2019 (COVID-19); prior studies further indicate that visceral obesity increases the risk of complications (2). Obesity, especially when accompanied by type 2 diabetes, is also a substantial risk factor for nonalcoholic liver disease (NAFLD) (3). Moreover, obesity has been identified as a risk factor for some types of malignancies, including kidney cancer, one of the most prevalent malignancies of the urinary system with increasing incidence (4–6).

Obesity and metabolic syndrome share numerous pathophysiological pathways (7). Accordingly, the significance of obesity as an independent contributor to adverse health outcomes, regardless of obesity-induced metabolic abnormalities, is still debatable. A subset of individuals with obesity without metabolic abnormalities, referred to as “metabolically healthy obesity” (MHO), has attracted academic interest since 2001 (8–10). Although some previous studies have reported the benign nature of the MHO phenotype, the clinical implication of MHO presents significant challenges, and its clinical value may vary depending on the study outcomes (8–11). To the best of our knowledge, evidence on the association between MHO and kidney cancer is lacking. In particular, it is uncertain whether obesity, apart from obesity-related metabolic abnormalities, plays a critical role in the development of kidney cancer. Furthermore, metabolic health and obesity phenotypes are not permanent, and changes in body weight or metabolic health status may shift an individual into a different group, resulting in different health outcomes (12). Thus, while discussing the impact of metabolic health and obesity on clinical outcomes, we must include phenotypic shifts over time.

In this context, this study aimed to evaluate the influence of transitions in metabolic health status over time on the kidney cancer risk in the obese Korean population using a large nationwide cohort.

## Materials and methods

### Study population

Data from the Korean National Health Insurance Service-National Health Screening Cohort (NHIS-HEALS) were utilized in this investigation. The Korean NHIS currently collects and administers databases on the usage of all health services in Korea (13). This cohort includes a total of 514,866 individuals who completed NHIS health screening tests and were randomly sampled. The detailed composition of this cohort was previously discussed in the previous literature (13). The index period was from January 1, 2009, to December 31, 2010, as some laboratory measurements, including TG and HDL-C, which are essential for evaluating metabolic health, were collected since 2009. Participants who died (n=24,593) or were diagnosed with kidney cancer (n=1,667) before the end of the index period were excluded from the analysis. Additionally, individuals with missing baseline data for blood pressure (BP), body mass index (BMI), fasting plasma glucose (FPG), and lipid levels were excluded. Finally, 321,124 participants were included in our study.

The NHIS Investigation Commission authorized the study. As this study used the collected data from NHIS-HEALS, no informed consent was acquired from each participant, and all data were thoroughly de-identified and anonymized. This study was approved by the Hallym Sacred Heart Hospital Institutional Review Board (IRB) (IRB No. 2021-02-001).

### Definitions of metabolic health and obesity status

Obesity was defined as BMI ≥25 kg/m^2^ using the Asia-Pacific standards developed by the World Health Organization’s Western Pacific Region (14–16). According to the Adult Treatment Panel III criteria, metabolic health was defined as having no more than one of the risk factors (17): (1) BP >130/85 mmHg or the use of antihypertensive drugs, (2) TG level >150 mg/dl or the use of lipid-lowering drugs, (3) HDL-cholesterol level <40 mg/dl (men) or 50 mg/dl (women), or (4) FPG level >100 mg/dl or the use of an anti-diabetic treatment. At the baseline examination, the study cohort was divided into the metabolically healthy non-obese (MHNO) group, MHO group, metabolically unhealthy non-obese (MUNO) group, and metabolically unhealthy obesity (MUO) group. According to the results from the next biannual examination, we categorized the obese participants into the stable MHO group, MHO to the MUO group, MUO to the MHO group, and stable MUO group.

### Definitions of kidney cancer and covariates

The study endpoint was kidney cancer diagnosis from the index date until the end of 2015. The diagnosis of kidney cancer was defined according to the International Classification of Diseases (ICD)-10-CM code (C64). Diabetes, hypertension, dyslipidemia, smoking habits, drinking habits, and physical activity were defined as previously described (18). We adjusted for baseline age, sex, smoking habits, drinking habits, physical activity, and estimated glomerular filtration rate level.

### Statistical analyses

Continuous data are presented as means ± standard deviation and categorical data as percentages. To analyze the baseline biochemical characteristics according to the metabolic health and obesity status, the analysis of the variance and the Scheffe’s test for *post hoc* analysis or the chi-squared test were adopted. Cox proportional hazards analysis was performed to calculate the hazard ratio (HR) and the 95% confidence interval (CI) of incident kidney cancer. Age, sex, smoking and drinking habits, physical activity, and eGFR levels were all factored into multivariate models. The risk of kidney cancer was first assessed based on the baseline obese metabolic health status within the MHNO group as the reference. Subsequently, the risk was analyzed further after considering the shift in metabolic health and obesity in participants with obesity at baseline. During the follow-up phase, the stable MHNO group was used as the reference group. P < 0.05 was considered statistically significant. The SAS Enterprise Guide software was used for all statistical analyses (version 7.1, SAS Institute, Inc., Cary, NC, USA).

## Results

### Baseline characteristics of the study population

**Table 1** displays the biochemical and clinical features of the patients at baseline, grouped by obesity categories and metabolic health status. The percentage of MHNO, MHO, MUNO, and MUO groups at baseline was 29.2%, 9.0%, 34.7%, and 27.2% of the entire cohort, respectively. Participants with MHO had a poorer lipid profile, including higher total cholesterol, LDL-C, and TG levels, and lower HDL-C values than participants with MHNO (all P <.0001). In contrast, the MHO group had lower FPG and TG levels and higher HDL-C levels than the MUNO group (all P <.0001). Male patients were more likely to be classified as metabolically unhealthy among the study participants.

**Table 1:** Characteristics of the study participants according to baseline metabolic health and obesity status.

Baseline category	MHNO	MHO	MUNO	MUO	P value
N	93,805 (29.2)	28,785 (9.0)	111,288 (34.7)	87,246 (27.2)	
Sex (% men)	48.6	50.4	56.0	58.3	<.0001
Age (yr)	57.5 ± 8.4^†^	57.5 ± 7.8^†^	60.0 ± 8.8	59.2 ± 8.3	<.0001
BMI (kg/m^2^)	22.1 ± 1.9	26.8 ± 1.6	22.7 ± 1.6	27.2 ± 1.9	<.0001
WC (cm)	77.1 ± 6.8	86.8 ± 6.5	80.4 ± 6.4	89.4 ± 6.6	<.0001
Systolic BP (mmHg)	119.0 ± 13.9	123.4 ± 14.0	129.6 ± 14.5	132.2 ± 14.2	<.0001
Diastolic BP (mmHg)	73.9 ± 9.3	76.4 ± 9.5	80.3 ± 9.5	82.0 ± 9.6	<.0001
Smoking (%)					<.0001
Current smoker	15.7	13.0	20.0	17.8	
Ex-smoker	15.9	18.8	19.3	22.9	
Non-smoker	68.4	68.2	60.6	59.3	
Drinking (%)					<.0001
None	61.7	59.4	57.1	54.4	
Mild	19.1	18.3	17.0	16.5	
Moderate	4.1	4.1	4.8	4.3	
Heavy	15.0	18.1	21.1	24.8	
Physical activity (%)					<.0001
None	26.9	27.9	29.6	29.8	
1–2 times/week	22.9	23.3	22.4	24.1	
3–4 times/week	22.0	21.5	21.3	21.4	
≥5 times/week	28.1	27.2	26.7	24.7	
Medical history (%)					
Type 2 diabetes	1.2	1.1	15.7	18.4	<.0001
HTN	13.1	22.1	44.7	55.8	<.0001
Dyslipidemia	3.8	4.1	29.4	34.8	<.0001
FPG (mg/dl)	91.6 ± 12.7	92.3 ± 11.9	108.3 ± 29.2	110.3 ± 29.3	<.0001
TG (mg/dl)	97.7 ± 46.0	108.9 ± 49.0	166.2 ± 101.7	188.0 ± 110.5	<.0001
LDL-C (mg/dl)	120.2 ± 33.6	125.9 ± 34.0	121.4 ± 41.0	122.6 ± 42.0	<.0001
HDL-C (mg/dl)	59.4 ± 25.6	56.8 ± 24.6	51.6 ± 27.5	49.2 ± 24.2	<.0001
TC (mg/dl)	198.4 ± 33.1	203.7 ± 33.7	203.8 ± 39.8	206.8 ± 39.9	<.0001
eGFR (mL/min/1.73m^2^)	82.7 ± 18.3	81.4 ± 18.0	78.6 ± 19.6	77.6 ± 19.5	

Results reported as means ± SD, unless otherwise indicated.

^†^No statistical difference was observed.

BMI, body mass index; BP, blood pressure; eGFR, estimated glomerular filtration rate; FPG, fasting plasma glucose; HDL-C, high-density lipoprotein cholesterol; HTN, hypertension; LDL-C, low-density lipoprotein cholesterol; MHO, metabolically healthy obesity; MUO, metabolically unhealthy obesity; MUNO, metabolically un-healthy obesity; MHNO, metabolically healthy non-obese; TC, total cholesterol; TG, triglyceride; WC, waist circumference.

### Incident kidney cancer according to metabolic health and obesity status

**Figure 1:**
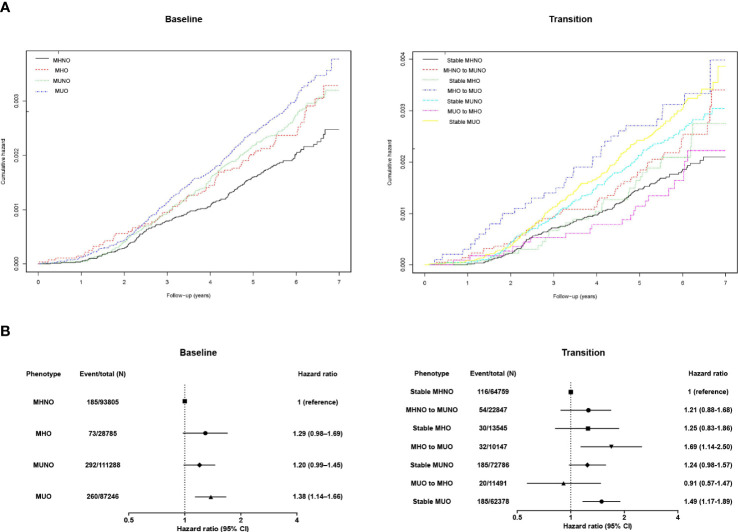
**(A)** Kaplan–Meier curves and the **(B)** hazard ratios (95% confidence intervals) for kidney cancer according to obese metabolic health status at baseline and in consideration of phenotypic transitions. The hazard ratios (95% confidence intervals) were adjusted for baseline age, sex, smoking habits, drinking habits, physical activity, and estimated glomerular filtration rate level. Abbreviations: MHNO, metabolically healthy non-obese; MHO, stable metabolically healthy obesity; MUO, metabolically unhealthy obesity; MUNO, metabolically unhealthy obesity.

**Figure 1A** depicts the Kaplan–Meier curves for the cumulative incidence of kidney cancer according to metabolic health and obesity status. MHO, MUNO, and MUO groups had higher probability of developing kidney cancer (log rank p<0.001). **Table 2** and **Figure 1B** describe the incident kidney cancer risk according to the obese metabolic health phenotype at baseline examination but do not consider the change over time. Compared with the MHNO group, only the MUO group had a 38% increased risk of incident kidney cancer after adjustment for age, sex, smoking habits, drinking habits, physical activity, and eGFR level (multivariate-adjusted HR, 1.38; 95% CI, 1.14–1.66). The risk of incident kidney cancer was not substantially greater in the MHO or MUNO groups than in the MHNO group.

**Table 2:** Risk of incident kidney cancer according to baseline metabolic health and obesity status.

Baseline category	MHNO	MHO	MUNO	MUO
N (% of total)	93,805 (29.2)	28,785 (9.0)	111,288 (34.7)	87,246 (27.2)
Number of events (%)	185 (0.20)	73 (0.25)	292 (0.26)	260 (0.30)
Crude HR (95% CI)	1 (ref)	1.28 (0.98–1.68)	1.33 (1.10–1.59)	1.50 (1.25–1.82)
Age and sex-adjusted HR (95% CI)	1 (ref)	1.28 (0.98–1.68)	1.22 (1.02–1.47)	1.40 (1.16–1.69)
Multivariate-adjusted HR (95% CI)^†^	1 (ref)	1.29 (0.98–1.69)	1.20 (0.99–1.45)	1.38 (1.14–1.66)

^†^Adjusted for baseline age, sex, smoking habits, drinking habits, physical activity, and estimated glomerular filtration rate level.

MHO, stable metabolically healthy obesity; MUO, metabolically unhealthy obesity; MUNO, meta-bolically unhealthy obesity; MHNO, metabolically healthy non-obese.

### Changes in the metabolic health status of the obese population and the risk of incident kidney cancer

Furthermore, we assessed the implication of phenotypic transitions on the risk of kidney cancer. Kaplan–Meier analyses showed that MHO to MUO transition and persistent MUO status are related to higher probability of kidney cancer (**Figure 1A**, log rank p<0.001). Then, we calculated the multivariate-adjusted HRs for incident kidney cancer considering transitions in the metabolic health status (**Table 3**; **Figure 1B**). The stable MHNO group was used as the referent group in the analysis. The participants with MUO at baseline and follow-up (i.e., the stable MUO group) had a considerably greater incidence of kidney cancer than the stable MHNO group (multivariate-adjusted HR, 1.49; 95% CI, 1.17–1.89). The participants who moved from MHO to MUO had a substantially greater risk of kidney cancer than did those in the reference group, with a multivariate-adjusted HR of 1.69 (95% CI, 1.14–2.50), although they were metabolically healthy at baseline. In contrast, neither the stable MHO group (multivariate-adjusted HR, 1.25; 95% CI, 0.83–1.86) nor the MUO to MHO group (multivariate-adjusted HR, 0.91; 95% CI, 0.57–1.47) had an elevated risk of incident kidney cancer. The MHNO to MUNO group and the stable MUNO group were not at increased risk of incident kidney cancer (multivariate-adjusted HR, 1.21; 95% CI, 0.88–1.68 and 1.24 (0.98–1.57), respectively). **Figure 1** depicts the multivariate-adjusted HRs for incident kidney cancer.

**Table 3:** Risks of incident kidney cancer according to the transition from metabolically healthy to unhealthy status among participants with obesity in reference to the stable MHNO group.

Dynamic category	Stable MHNO(reference)	MHNO to MUNO	Stable MHO	MHO to MUO	Stable MUNO	MUO to MHO	Stable MUO
N (% of respective baseline category)	64,759 (69.0)	22,847 (24.4)	13,545 (47.1)	10,147 (35.3)	72,786 (65.4)	11,491 (13.2)	62,378 (71.5)
Number of events (%)	116 (0.18)	54 (0.24)	30 (0.22)	32 (0.32)	185 (0.25)	20 (0.17)	185 (0.30)
Crude HR (95% CI)	1 (ref)	1.32 (0.96-1.82)	1.24 (0.83-1.85)	1.75 (1.19-2.60)	1.41 (1.12-1.78)	0.97 (0.60-1.55)	1.65 (1.31-2.08)
Age and sex-adjusted HR (95% CI)	1 (ref)	1.23 (0.89-1.70)	1.24 (0.83-1.85)	1.69 (1.14-2.50)	1.27 (1.01-1.61)	0.92 (0.57-1.48)	1.52 (1.20-1.92)
Multivariate-adjusted HR (95% CI)^†^	1 (ref)	1.21 (0.88-1.68)	1.25 (0.83-1.86)	1.69 (1.14-2.50)	1.24 (0.98-1.57)	0.91 (0.57-1.47)	1.49 (1.17-1.89)

^†^Adjusted for baseline age, sex, smoking habits, drinking habits, physical activity, and estimated glomerular filtration rate level.

MHO, stable metabolically healthy obesity; MUO, metabolically unhealthy obesity; MUNO, meta-bolically unhealthy obesity; MHNO, metabolically healthy non-obese.

### Subgroup analyses

**Figure 2:**
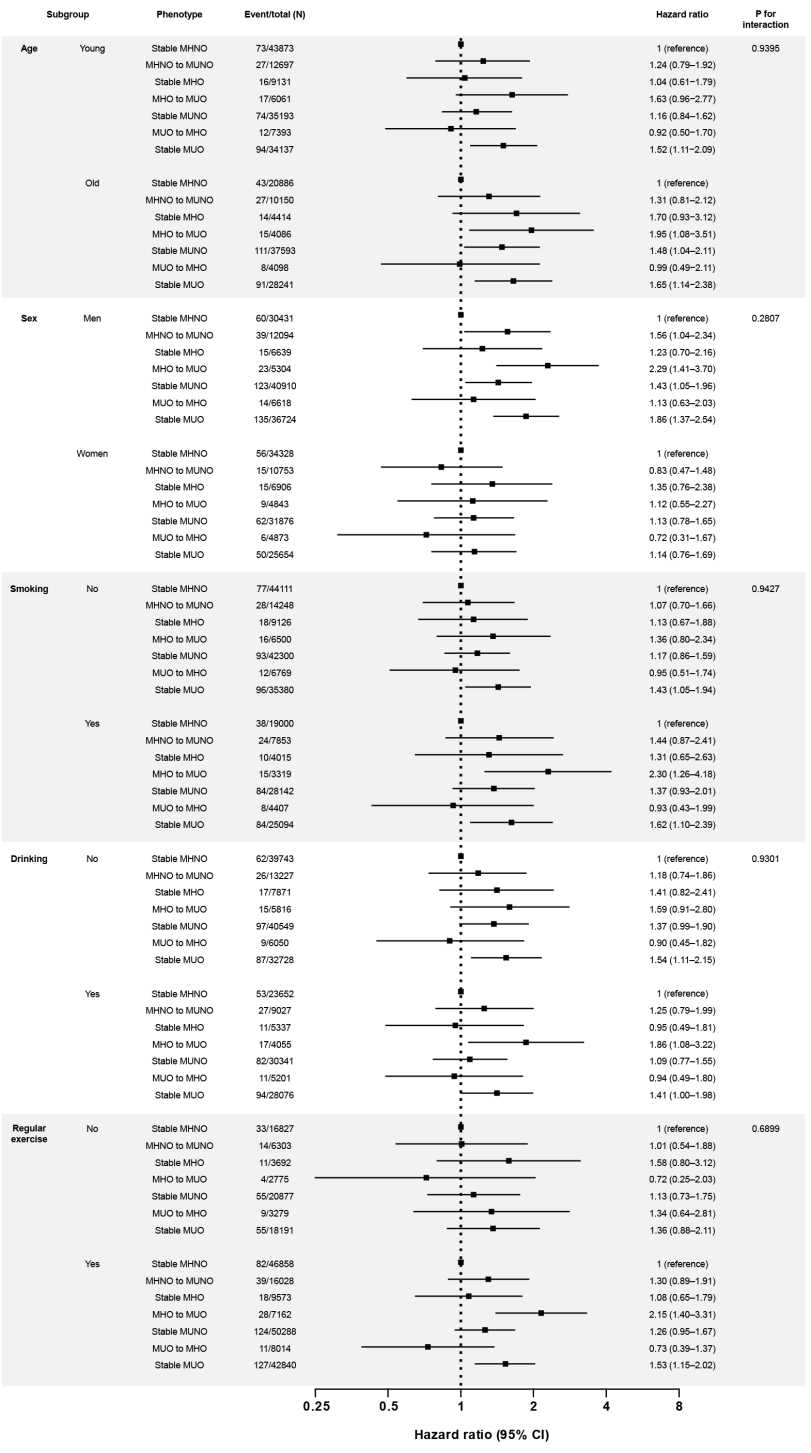
Subgroup analyses for the risk of kidney cancer according to the phenotypic transitions. The hazard ratios (95% confidence intervals) were adjusted for baseline age, sex, smoking habits, drinking habits, physical activity, and estimated glomerular filtration rate level. The covariates are excluded from the adjustment in the corresponding subgroup analyses. Abbreviations: MHNO, metabolically healthy non-obese; MHO, stable metabolically healthy obesity; MUO, metabolically unhealthy obesity; MUNO, metabolically unhealthy obesity.

Associations of the obese metabolic health phenotypes with kidney cancer were generally consistent across the subgroups according to the clinical variables, including age, sex, smoking, drinking, and exercise (**Figure 2**). In specific, the hazardous effect of phenotypic transition from MHO to MUO was particularly evident in men and smokers (multivariate-adjusted HR, 2.29; 95% CI, 1.41–3.70 in men; multivariate-adjusted HR, 2.30; 95% CI, 1.26–4.18 in smokers). Across all subgroups, obese participants who stayed at metabolically heath status (i.e., stable MHO groups) were not at increased risk of kidney cancer (**Figure 2**).

## Discussion

This study suggests that metabolic unhealthiness could contribute to kidney cancer risk in obese patients. We found that maintaining or recovering metabolic health reduced the incidence of kidney cancer, whereas the persistence of a metabolically unhealthy status or the shift to metabolic unhealthiness substantially increased the risk of kidney cancer. Our findings indicate that metabolic unfitness, rather than the presence of obesity, contributes to incident kidney cancer.

Previously, a dose-response meta-analysis reported that every 1 kg/m^2^ increase in BMI led to a 6% increase in HR for kidney cancer (6), whereas another systematic review and meta-analysis on the association between BMI and oncologic outcomes in patients with kidney cancer reported better survival in obese kidney cancer patients, supporting an obesity paradox (19). More recently, an increased risk of kidney cancer due to obesity has been consistently reported. In Japan, a population-based study demonstrated a U-shaped association between BMI and the risk of renal cell cancer (RCC) (20). Moreover, a population-based nested case-control study reported a positive relationship between BMI and the risk of RCC among Chinese men; this study showed an increased odds ratio of 1.5 (95% CI, 1.1–2.0) for a 5-kg/m^2^ increase in BMI (21). Positive linear relationships were found in the South Korean population between BMI (or waist circumference) and the risk of incident kidney cancer (22). In particular, individuals with both general obesity and central obesity had a 1.45-fold increase in the risk of incident kidney cancer, which exceeded the 1.32-fold increase of general or central obesity (22). These studies support the significant implication of obesity in the risk of kidney cancer. However, these studies did not take metabolic health status into consideration.

Here, we found that the incident kidney cancer risk among obese individuals depended on their metabolic health status. Based on the baseline metabolic health status, the HR for kidney cancer in the MHO group was not significantly higher than that in the MHNO group (**Table 2**; **Figure 1**). However, when the phenotypic transition was considered, the probability of incident kidney cancer was significantly higher in individuals who were in the MHO group at baseline but transitioned to an MUO status and in those who maintained a steady MUO phenotype (**Table 3**, **Figure 1**). In contrast, the stable MHO group or the MUO to MHO group were not at a higher risk of developing kidney cancer even though they were still obese (**Table 3**; **Figure 1**), which were consistently observed in subgroup analyses (**Figure 2**). These data imply that metabolic health, not obesity itself, is a decisive factor in kidney cancer incidence. Previously, in the MetS and cancer project, several metabolic factors or a combination of risk factors were found to be associated with an increased risk of RCC (23). Similarly, a nationwide study in Korea reported that MetS was closely related to the risk of kidney cancer in both sexes; specifically, patients with MetS had significantly increased HRs for incident kidney cancer, and this relationship was consistent in both men and women (men: HR, 1.32; 95% CI, 1.25–1.40; women: HR, 1.39; 95% CI, 1.25–1.53) (24). Collectively, metabolic disturbances induced by disproportional body fat distribution could be the main contributor to incident kidney cancer in participants with obesity.

In our study, we suggested that the metabolic health status was a largely modifiable risk factor. Prior studies have reported that approximately one-third of individuals with obesity experienced changes in their metabolic health status (25–28), potentially affecting their health outcomes. Therefore, recent studies have adopted novel approaches to reflect the influence of phenotypic transitions on diverse outcomes. For example, Kim et al. have discovered that maintaining metabolic fitness could protect the study participants from developing type 2 diabetes, regardless of their body weight (29). Moreover, our research team discovered that phenotypic alterations in MHO increased cardiovascular risk, CKD incidence, and mortality (25, 26). More recently, we demonstrated that metabolic health status was a deciding factor for the occurrence of colorectal cancer, for which obesity was known as a major risk factor (18). Herein, we added another evidence that we should consider the dynamic nature of metabolic health status in risk assessment and management in obese patients.

Although the specific mechanism through which obesity raises the risk of kidney cancer is yet to be determined, the altered circulating levels of adipokines (30), the chronic inflammatory status (31), and modulation of host immunosurveillance (30), and insulin resistance leading to increased insulin and insulin-like growth factor (IGF)-1 levels, which are involved in carcinogenesis may play a significant role (6, 32, 33). Although our results cannot establish the mechanism, our data provide evidence that metabolic unhealthiness associated with obesity plays a pivotal role in the increased risk of kidney cancer in patients with obesity. Therefore, further investigations on the pathophysiologic changes in different metabolic health obese phenotypes are needed.

This study had some limitations. First, since the study population was primarily Korean, we cannot generalize our study results to other ethnic groups. Second, the study did not consider the phases of kidney cancer or its pathologic type. Thirdly, an accurately measured increase in lower body fat mass is now recognized as an independent indicator of metabolic health (34). Therefore, the identification of distinct fat distribution phenotypes using relevant measurements, such as hip circumference, could provide better insight into the relationship between adiposity and cancer risk; however, we were unable to investigate the impact of these measurements on KC risk in our analyses because the NHIS data did not include any measurement for lower body fat mass. Future study on the significance of lower body fat mass in obesity-related cancer would give greater precision to our understanding of the clinical implications of metabolic health in obese populations. Despite these limitations, our study has strengths in that we used a large nationwide cohort and explained the effects of dynamic metabolic health on the incidence of kidney cancer in obese adults. Our methodology revealed the implication of metabolic unhealthiness on kidney cancer risk and therefore suggested that being metabolically healthy should be prioritized to lower the kidney cancer risk in obese patients.

## Conclusions

Our findings identified metabolic unhealthiness as a risk factor for kidney cancer risk in individuals with obesity. Furthermore, our results suggest that the dynamic metabolic health status should be considered as significantly affecting the kidney cancer risk. Therefore, while assessing the association between obesity and kidney cancer, physicians should examine patients’ metabolic health conditions and educate them on the necessity of metabolic fitness.

## Data availability statement

Anonymized data are publicly available from the National Health Insurance Sharing Service and can be accessed at https://nhiss.nhis.or.kr/bd/ab/bdaba000eng.do, approval number: NHIS-2021-2-219.

## Ethics statement

The studies involving human participants were reviewed and approved by Hallym Sacred Heart Hospital Institutional Review Board (IRB). The ethics committee waived the requirement of written informed consent for participation.

## Author contributions

Conceptualization, YC and CJ; methodology, Y-JK; software, Y-JK; validation, YC and Y-JK; formal analysis, YC and Y-JK; investigation, YC; resources, YC; data curation, CJ; writing—original draft preparation, YC; writing—review and editing, HK, J-YP, WL, Y-JK, and CJ; visualization, Y-JK; supervision, YC. All authors have read and agreed to the published version of the manuscript.

## Funding

This research was supported by the Hallym University Research Fund 2021 (HURF-2021-19).

## Acknowledgments

The authors thank Editage for the English language review. We would like to thank the Korean National Health Insurance Service and all the participants of the study and health check-up.

## Conflict of interest

The authors declare that the research was conducted in the absence of any commercial or financial relationships that could be construed as a potential conflict of interest.

## Publisher’s note

All claims expressed in this article are solely those of the authors and do not necessarily represent those of their affiliated organizations, or those of the publisher, the editors and the reviewers. Any product that may be evaluated in this article, or claim that may be made by its manufacturer, is not guaranteed or endorsed by the publisher.

## Abbreviations

BMI, body mass index; BP, blood pressure; FPG, fasting plasma glucose; ICD, International Classification of Diseases; IRB, Hallym Sacred Heart Hospital Institutional Review Board; MHNO, metabolically healthy non-obese; MHO, metabolically healthy obesity; MHO, metabolically healthy obesity; MUNO, metabolically unhealthy non-obese; MUO, metabolically unhealthy obesity; NHIS-HEALS, Korean National Health Insurance Service-National Health Screening Cohort; RCC, renal cell cancer.
